# Substitution of outpatient hospital care with specialist care in the primary care setting: A systematic review on quality of care, health and costs

**DOI:** 10.1371/journal.pone.0219957

**Published:** 2019-08-01

**Authors:** Sofie J. M. van Hoof, Tessa C. C. Quanjel, Mariëlle E. A. L. Kroese, Marieke D. Spreeuwenberg, Dirk Ruwaard

**Affiliations:** 1 Department of Health Services Research, Care and Public Health Research Institute (CAPHRI), Faculty of Health Medicine and Life Sciences, Maastricht University, Maastricht, the Netherlands; 2 Research Centre for Technology in Care, Zuyd University of Applied Sciences, Heerlen, the Netherlands; Osakidetza Basque Health Service, SPAIN

## Abstract

**Rationale, aims and objective:**

Substituting outpatient hospital care with primary care is seen as a solution to decrease unnecessary referrals to outpatient hospital care and decrease rising healthcare costs. This systematic review aimed to evaluate the effects on quality of care, health and costs outcomes of substituting outpatient hospital care with primary care-based interventions, which are performed by medical specialists in face-to-face consultations in a primary care setting.

**Method:**

The systematic review was performed using the PICO framework. Original papers in which the premise of the intervention was to substitute outpatient hospital care with primary care through the involvement of a medical specialist in a primary care setting were eligible.

**Results:**

A total of 14 papers were included. A substitution intervention in general practitioner (GP) practices was described in 11 papers, three described a joint consultation intervention in which GPs see patients together with a medical specialist. This study showed that substitution initiatives result mostly in favourable outcomes compared to outpatient hospital care. The initiatives resulted mostly in shorter waiting lists, shorter clinic waiting times and higher patient satisfaction. Costs for treating one extra patient seemed to be higher in the intervention settings. This was mainly caused by inefficient planning of consultation hours and lower patient numbers.

**Conclusions:**

Despite the fact that internationally a lot has been written about the importance of performing substitution interventions in which preventing unnecessary referrals to outpatient hospital care was the aim, only 14 papers were included. Future systematic reviews should focus on the effects on the Triple Aim of substitution initiatives in which other healthcare professions than medical specialists are involved along with new technologies, such as e-consults. Additionally, to gain more insight into the effects of substitution initiatives operating in a dynamic healthcare context, it is important to keep evaluating the interventions in a longitudinal study design.

## Introduction

The Organisation for Economic Co-operation and Development (OECD) has stated that the pressure on healthcare systems is increasing worldwide, since healthcare costs are rising and the sustainability of healthcare systems is therefore at stake [1]. The International Monetary Fund (IMF) has stated that spending on health care is one of the key drivers in the increasing total spending of countries over the past 40 years [2]. The World Health Organization (WHO) has estimated that 20% to 40% of healthcare spending is wasted through inefficient use of health care [3]. It is assumed that reforming healthcare systems with a view to making better use of resources will make a key contribution to decreasing spending on health care. There are many ways in which increased efficiency could be accomplished, for example by motivating healthcare workers, reducing medical errors by providing care at the right moment and place, eliminating waste and corruption, assessing critically what care services are needed and improving hospital efficiency by preventing unnecessary hospital attendances [3].

Many governments stimulated to start interventions that are aimed at providing healthcare at the right time and the right place in order to contain rising costs, without compromising on quality and health of the patients [3]. Outpatient hospital care is more expensive than primary care, and unnecessary attendances should therefore be prevented [4]. According to Berwick et al., when performing new interventions to achieve positive healthcare reforms, interventions should have positive effects on the Triple Aim [5]. High-value health care can only be accomplished if new interventions pursue an improvement of the individual experience of care and health of the involved population and a reduction of the costs per capita.

In disease management programmes attention is given to providing care for chronic patients at the right time and place to improve the quality of care and to reduce healthcare costs [6]. Bundled payment systems are becoming more and more part of integrated care programmes to support the reduction of healthcare costs [7–10]. Many systematic reviews have focused on the effects of integrated care programmes for disease-specific programmes on quality of care, effectiveness and efficiency gain [11, 12]. There are reviews on the effects of (specialist) outreach clinics, a specific type of intervention, on the aims of the Triple Aim [13, 14] and reviews in which substitution initiatives, in a broad sense, and their effects on one or two elements of the Triple Aim are studied [15, 16]. Our current study, however, is aimed at substitution initiatives that focus on medical specialists who provide face-to-face consultations in the primary care setting. Substitution of care can be defined as ‘the continual regrouping of resources across and within care settings to exploit the best and least costly solution in the face of changing needs and demands’ [17]. Additionally, various types of substitution consist [17]. This review is aimed at one of these types, namely moving the location at which care is given. Moreover, this review is specifically focused on moving the location of specialist care towards the primary care setting. The objective of this literature review was to report on primary care-based interventions where medical specialists provide face-to-face consultations in a primary care setting and the effects of these interventions on Triple Aim related outcomes.

## Materials and methods

### Search strategy

In this study a systematic search in two electronic databases (PubMed and Cochrane Library) was conducted, focused on papers published between 1 January 1985 and 16 March 2017. This time frame was chosen because an initial quick search showed that many studies on the effects of substitution interventions were conducted in the late eighties and early nineties. Additional papers were tracked via references of included papers or relevant systematic reviews. No review protocol was used for this systematic review.

The search strategy was created using the PICO framework [18]. The patients or profession (P) used in the search strategy were indicated by the setting in which the intervention was supposed to take place or the professionals involved (primary care, family practice or general practitioners (GPs)). An initial quick search was performed to create the intervention section of the search strategy. The interventions (I) were indicated as joint consultations, outreach services or clinics, specialist service, substitution or visiting service. The definition of controls (C) was omitted, because we wanted also to include studies without a control group. The outcomes (O) were related to the Triple Aim (quality of care, health outcomes or healthcare costs). At least one of the Triple Aim outcomes was supposed to be measured in the study. Quality of care could be operationalized according to the guidelines of the Institute of Medicine (IOM) on measuring quality of care in terms of safety of care, effectiveness, patient-centredness, timeliness, efficiency and/or equitability [19]. Health could be measured in different ways, e.g. as experienced health, disease burden or quality of life. Costs could be measured as direct and/or indirect costs (e.g. number of hospital visits, overhead costs, per capital costs of care). The full search strategy can be found in S1 File.

Original papers in which the premise of the intervention was to substitute outpatient hospital care with primary care through medical specialists who provide face-to-face consultations in the primary care setting were eligible for this review. Patients involved in the studied initiatives should be aged 18 or older. Medical complaints should be non-acute, non-dental and non-mental. The interventions, in which face-to-face consultations are performed, should be carried out in OECD high-income countries, according to the list of the World Bank [20]. Finally, papers should be written in English or Dutch.

### Relevance

Two researchers (SJMH and TCCQ) made an initial selection based on titles and abstracts. Afterwards, discrepancies were resolved through discussion between reviewers. All relevant studies and studies where there was any doubt were read in full and reviewed independently by SJMH and TCCQ with a view to final inclusion (or exclusion). In the case of disagreement, a third reviewer (MEALK) was consulted to reach consensus.

### Quality screening

The quality of included papers was assessed by two researchers (SJMH and TCCQ) independently using the quality assessment tool for quantitative studies of the Effective Public Health Practice Project [21]. The checklist consisted of six components: 1) selection bias; 2) study design; 3) confounders; 4) blinding; 5) data collection methods; and 6) withdrawals and dropouts. Each component was subdivided into two to four subcomponents. Each component was given an overall score of ‘strong’, ‘moderate’ or ‘weak’. Finally, a global rating for the paper was given, based on the six components.

#### 1) Selection bias

This component entailed the representativeness of the individuals selected to participate in the study and the percentage of the selected individuals that agreed to participate. If it was likely that the study population was representative and a large percentage of individuals agreed to participate, the section was rated as strong.

#### 2) Study design

This component could be rated as strong if the study was a randomized controlled trial (RCT) or a controlled clinical trial (CCT). The quality assessment tool describes an RCT as an experimental study in which the investigators randomly allocate patients to an intervention or control group; patients have the same chance of being allocated to one of the groups. A CCT is described as an experimental study in which the method of allocation of patients is open to the people responsible for recruiting patients; the method of allocation is transparent before assignment. The rate was moderate if the study was a cohort analytic study, a case control study, a cohort design or an interrupted time series. A cohort analytic study is an observational study design in which groups are assembled according to whether or not they have been exposed to the intervention, and the groups are tested pre and post. A case control study is a retrospective study design in which the researchers gather patients who already have the outcome of interest, and control patients who do not. Both groups are tested post intervention. A cohort study is described as a study design in which one group is pre and post tested. Finally, the interrupted time series is described as a study design in which multiple observations are tested over time. Study designs other than those described above were rated as weak.

#### 3) Confounders

This component could be rated as strong if the allocation to intervention and control groups is randomized—which means the groups were balanced at baseline with respect to confounders—or if there were any confounders described and most confounders were controlled for. If some or no confounders were controlled for, the study had to be rated either as moderate or weak, respectively.

#### 4) Blinding

If outcome assessors were aware of the exposure status of participants and participants were aware of the research question, the study had to be rated as weak. If in both cases the answer was no, the study was rated as strong. If one of the subcomponents was true, the study was rated as moderate.

#### 5) Data collection methods

If data collection tools were judged to be valid and reliable, the study was rated as strong. If only one of the subcomponents was positively evaluated, the study was rated as moderate. If both subcomponents were shown not to be true, the study was rated as weak.

#### 6) Withdrawals and dropouts

If the percentage of participants that completed the study was high (between 80 and 100%) this component was rated as strong. The study was rated as moderate if the percentage was between 60 and 79% and a percentage lower than 60 was rated as weak.

The overall score was measured as follow: when there were no weak ratings, the paper could be scored as ‘strong’. When there was one weak rating or when there were no weak ratings but more than two moderate ratings, the paper could be scored as ‘moderate’. And finally, when there were two or more weak ratings, the paper could be scored as ‘weak’.

### Extraction of data

Due to heterogeneity of the study designs, outcome measures and data of the included papers, only descriptive synthesis was possible.

## Results

**Fig 1:**
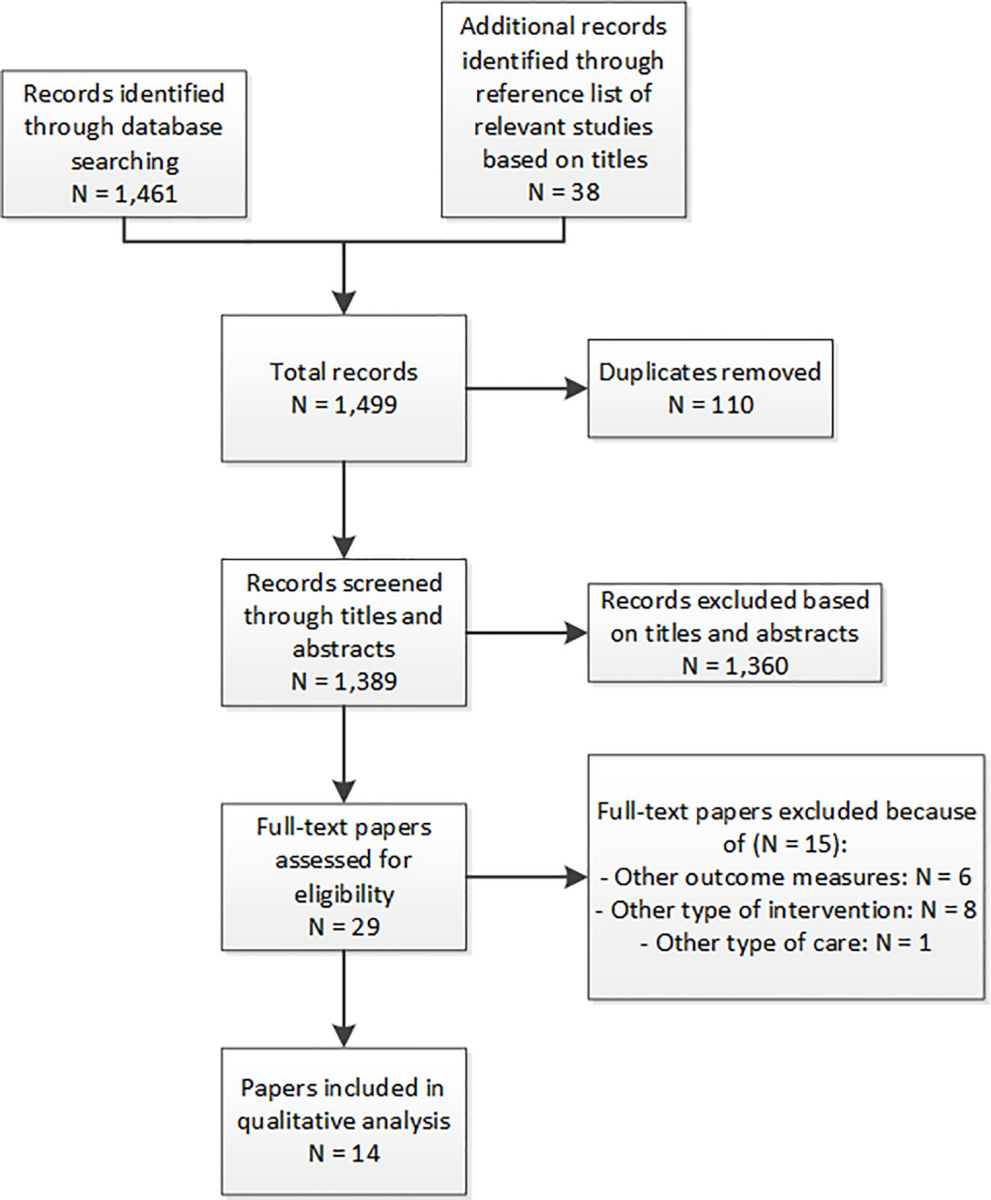
Flowchart of the searching and selection process.

Fig 1 shows a flowchart, according to the Prisma guidelines for systematic reviews (see S2 File), of the searching and selection process of relevant papers for this review [22]. An initial search resulted in 1,461 records. An additional search of reference lists of relevant papers, based on titles, and relevant systematic reviews resulted in 38 additional records. After removing duplicates, 1,389 papers remained, whose titles and abstracts were screened. Deliberation between researchers SJMH and TCCQ showed that they agreed on 96% of the titles and abstracts with regard to inclusion, exclusion or doubt. Further deliberation took place on the titles and abstracts to reach consensus on 100% of the papers. Given the extensive search criteria necessary for the definition of relevant interventions and many outcome measures, many papers were irrelevant for this review.

After reading the full-text of 29 papers, 14 appeared to be relevant for the review process. The majority of included papers originated from the United Kingdom (n = 10), three were from the Netherlands and one from Spain. Of these papers, 11 described an intervention in which medical specialists performed consultations in GP practices, and three described a joint consultation intervention in which GPs saw patients (cases) together with a medical specialist.

Descriptions of the 14 papers and characteristics of the studied interventions are summarised in Table 1 (an extended version of Table 1 can be found in S2 Table). Results of the 14 papers are described in Table 2, clustered per type of intervention. The outcome measurements and results are described per outcome of the Triple Aim.

**Table 1:** Characteristics of 14 included papers in alphabetical order.

Author(s), year	Country	Type of intervention	Medical specialty(ies) involved	Study design	Sample characteristics			Control group
					N	Age (mean)	Gender (% male)	
Black et al., 1997 [23]	United Kingdom	Substitution intervention in a (multidisciplinary) GP practice (outreach clinics)	Dermatology and orthopaedics	Cohort analytic study	164 patients 6 medical specialists 6 GPs	x	x	Patients in outpatient hospital care
Bond et al., 2000 [24]	United Kingdom	Substitution intervention in a (multidisciplinary) GP practice (outreach clinics)	Cardiology, ENT, general medicine, general surgery, gynaecology and rheumatology	Cohort analytic study	1420 patients 18 medical specialists 54 GPs	7% < 16 4% 16–25 31% 25–45 35% 45–65 23% ≥ 65	32	Patients in outpatient hospital care
Bowling and Bond, 2001 [25]	United Kingdom	Substitution intervention in a (multidisciplinary) GP practice (outreach clinics)	Cardiology, ENT, general medicine, general surgery, gynaecology, paediatrics and rheumatology	Cohort analytic study	2925 patients 38 medical specialists 196 GPs	9% < 16 5% 16–25 30% 25–45 33% 45–65 23% > 65	31	Patients in outpatient hospital care
Bowling et al., 1997 [26]	United Kingdom	Substitution intervention in a (multidisciplinary) GP practice (outreach clinics)	ENT, rheumatology and gynaecology	Cohort analytic study	146 patients 9 practice managers 9 medical specialists 60 GPs	x	x	Patients in outpatient hospital care
Dart, 1986 [27]	United Kingdom	Substitution intervention in a (multidisciplinary) GP practice	Ophthalmology	Cohort study	46 patients	x	x	Diagnosis of GP was compared to diagnosis of ophthalmologist
Gillam et al., 1995 [28]	United Kingdom	Substitution intervention in a (multidisciplinary) GP practice (outreach clinics)	Ophthalmology	Cohort analytic study	1309 patients 63 GPs	x	x	Patients in outpatient hospital care
Gosden et al., 1997 [29]	United Kingdom	Substitution intervention in a (multidisciplinary) GP practice (outreach clinics)	Dermatology and orthopaedics	Cohort analytic study	242 patients	x	x	Patients in outpatient hospital care
Helliwell, 1996 [30]	United Kingdom	Substitution intervention in a (multidisciplinary) GP practice (rotating community clinic)	Rheumatology	Cohort analytic study	135 patients	x	30.4	Patients in outpatient hospital care
Little et al., 1993 [31]	United Kingdom	Substitution intervention in a (multidisciplinary) GP practice (outreach clinics)	Ophthalmology	Cohort study	126 patients aged 75 and older	x	x	x
Schulpen et al., 2003 [34]	the Netherlands	Joint consultation	Rheumatology	Randomized controlled trial	166 patients 6 medical specialists 17 GPs	53.7	27	Patients in outpatient hospital care
Sibbald et al., 2008 [32]	United Kingdom	Substitution intervention in a (multidisciplinary) GP practice	Dermatology, ENT, general surgery, gynaecology, orthopaedics and urology	Cohort analytic study	58 service managers, GPs, care providers and medical specialists 1,233 Patients	x	x	Patients in outpatient hospital care
Surís et al., 2007 [35]	Spain	Joint consultation	Rheumatology	Cohort study	120 consultancy session cases 117 patients	x	x	x
Van Hoof et al., 2016 [33]	the Netherlands	Substitution intervention in a (multidisciplinary) GP practice (Primary Care Plus)	Internal medicine, dermatology, orthopaedics and neurology	Case control study	78 patients in intervention group 104 patients in control group	Intervention group 54.7 Control group 53.8	Interven-tion group 41.5 Control group 38.6	Patients in outpatient hospital care
Vierhout et al., 1995 [36]	the Netherlands	Joint consultation	Orthopaedics	Randomized controlled trial	272 patients	Intervention group 16% > 60 years Control group 13% > 60 years	Interven-tion group 49 Control group 50	Patients in outpatient hospital care

Abbreviations: GP = general practitioner, ENT = Ear, nose and throat

**Table 2:** Summary of results of included papers.

**Author, year**	**Outcome measurement**	**Results**
**Substitution interventions in a (multidisciplinary) GP practice**		
**Quality of care**		
Black et al., 1997 [23]	Quality outcomes and patient satisfaction measured via Group Health Association of America Consumer Satisfaction Survey a. Waiting time patient b. Travelling time patient c. Waiting time in clinic d. Patient satisfaction	a. Lower waiting times for first appointment in dermatology outreach clinic (69 days) compared to outpatient hospital care (97 days) b. Lower median travelling time for dermatology outreach clinic (20 min) compared to outpatient hospital care (40 min) c.—Higher median waiting time in dermatology outreach clinic (30 min) compared to outpatient hospital care (15 min) - Lower median waiting time in orthopaedics outreach clinic (10 min) compared to outpatient hospital care (25 min) d.—Satisfaction is higher in dermatology outpatient hospital care compared to outreach clinic for time spent waiting at clinic to see specialist - Satisfaction is higher in orthopaedics outreach clinic compared to outpatient hospital care for: location of clinic, length of consultation, time spent waiting at clinic, and specialists’ explanation of what was done
Bond et al., 2000 [24]	Self-administered patient questionnaire (Davies and Ware’s Visit-Specific Patient Satisfaction Survey). Outcome measurements for: a. Waiting list times b. Waiting times at clinic c. Follow-up after consultation d. Patient satisfaction with clinic	a. Outreach clinics had an average waiting list of 5.4 weeks compared to 7.8 weeks for outpatient hospital care b. Outreach clinics had shorter waiting times in clinics on average (14.4 min) compared to outpatient hospital care (29.9 min) c. More outreach patients were completely discharged after consultation compared to outpatients d. Outreach patients were more satisfied with all but one of the process and quality issues asked about in the survey. E.g. they were more satisfied with the convenience of the location of the clinic and the characteristics of the medical specialist
Bowling and Bond, 2001 [25]	Self-administered questionnaire for patients (not clear what kind of questionnaire). Outcome measurements on: a. Waiting list times b. Waiting times at clinic c. Follow-up after consultation d. Patient satisfaction with clinic	a. Outreach clinics had an average waiting list of 5.7 weeks compared to 7.9 weeks for outpatient hospital care b. Outreach clinics had shorter waiting times in clinics on average (15.9 min) compared to outpatient hospital care (32.8 min) c. Outreach patients were more likely to be discharged after a consultation than outpatients d. Satisfaction with the clinic was higher among outreach patients compared to outpatients. Satisfaction was greatest in relation to convenience of the clinic’s location, the clinic’s waiting area and the waiting time in the clinic
Bowling et al., 1997 [26]	Self-administered patient questionnaire (Davies and Ware’s Visit-Specific Patient Satisfaction Survey). Outcome measurements for: a. Waiting list times b. Waiting times at clinic c. Follow-up after consultation d. Patient satisfaction with clinic	a. No significant differences in waiting list times overall. For gynaecology 53% of outreach patients waited less than 3 weeks compared to 15% of outpatients. b. 33% of outreach patients waited 10 min or less compared to 12% of outpatients. Outpatients were more likely to wait for an hour or more (22%) than outreach patients (5%) c. 37% of outreach patients needed follow-up compared to 50% of outpatients d. Outreach patients were more satisfied than outpatients with the length of the waiting time at the clinic, the amount of time spent with the medical specialist, the convenience of the appointment, the clinic’s waiting area and attention given to what the patient had to say
Dart, 1986 [27]	-	-
Gillam et al., 1995 [28]	Self-administered questionnaire for patients and GPs (no description of types of questionnaires). Outcome measures were: a. Reported ability of GPs to diagnose and manage 14 ophthalmic conditions and changes in their referral policy b. Travel distances c. Journey times d. Waiting times e. Views on the service	a. GPs who spent time with the specialist for learning opportunities felt better able to manage one or more of the 14 conditions than those GPs who did not spend time with the medical specialist b. 22% of outpatients needed to travel more than 5 miles compared to 1.2% outreach patients c. No outreach patients needed to travel more than 50 min compared to 12% of the outpatients d. 94.9% of the outreach patients were helped within 30 min compared to 86.0% of the outpatients e. The majority of patients in both groups were satisfied with the service provided. No differences between the two groups
Gosden et al., 1997 [29]	Patient satisfaction questionnaire is used, no description of which questionnaire Outcome measurements for: a. Waiting times b. Satisfaction issues	a. Dermatology outreach patients had shorter waiting times compared to hospital patients (median of 69 and 97 days, respectively). No significant differences in waiting times for orthopaedic patients b. The results of the patient satisfaction indicated that the interpersonal nature of the consultation itself was more important to patients than issues of access or convenience, although the extent to which these findings can be generalized is not known
Helliwell, 1996 [30]	Survey of patient views by Kirklees Community Health Council. Outcome measurements: a. Travel distance b. Timeliness of appointment c. Quality of appointment d. Understanding of doctor e. Follow-up	a. The mean distance for community clinic (CC) patients was much lower than for hospital clinic (HC) patients (1.62 vs 4.98 miles, respectively) b. More CC patients saw the specialist at the appointment time (94%) compared to HC patients (71%) c. 82% of CC patients answered ‘always’ on quality questions compared to 52% of HC patients d. 85% of CC patients said that the doctor understood their problems compared to 53% of HC patients e. 64% of CC patients were discharged after the first consultation compared to 50% of HC patients
Little et al., 1993 [31]	No description of which questionnaire to measure quality a. Follow-up	a. Hospital follow-up for 16% of the patients screened in the community clinic. 29% of patients screened by GPs were recommended for hospital follow-up
Sibbald et al., 2008 [32]	Patient questionnaire based on Parchman et al. (2005). The survey covered the domains of service access, quality of care and coordination of care Outcome measurements on: a. Waiting time b. Access c. Coordination of care d. Interpersonal quality of care e. Technical quality of care f. Overall satisfaction	Only a. resulted in significant outcomes for the relocation service compared to conventional services (outpatient hospital care). The waiting time for relocation was 6.7 weeks compared to control services (10.1 weeks).
Van Hoof et al., 2016 [33]	Patient satisfaction was measured using an extraction of the Consumer Quality Index. Outcome measurements on: a. Information given by the medical specialist b. Cooperation between medical specialist and the institution (Primary Care Plus or hospital) c. Result of the treatment d. Medical specialist e. Institution (Primary Care Plus or hospital) g. Follow-up after consultation in Primary Care Plus was measured using a medical specialist questionnaire	a. Only the information given by the medical specialist had a significantly higher outcome for the intervention group (8.3) compared to the control group (7.8) g. Follow-up consultations in outpatient hospital care were necessary in 21.9% of the cases
**Author, year**	**Outcome measurement**	**Results**
**Health**		
Black et al., 1997 [23]	a. Health status measured via the Health Status Questionnaire-12 (HSQ-12) b. For dermatology patients the Dermatology Life Quality Index (DLQI) was also used A postal survey was sent 3 months after visiting one of the clinics	a. Significant greater improvement of health status of dermatology outpatients at 3-month follow-up compared to outreach patients a. No significant differences in health status of orthopaedic patients at 3-month follow-up
Bond et al., 2000 [24]	Health status was measured via the HSQ-12	Outreach patients had a significantly slightly better health status at follow-up compared to outpatients
Bowling and Bond, 2001 [25]	Health status was measured via the HSQ-12	Outreach patients had a higher health perception at follow-up than outpatients. And outreach patients scored higher in pain perceptions compared to outpatients at follow-up
Bowling et al., 1997 [26]	Health was measured using the RAND and SF-36	No health outcomes were described
Dart, 1986 [27]	-	-
Gillam et al., 1995 [28]	-	-
Gosden et al., 1997 [29]	-	-
Helliwell, 1996 [30]	-	-
Little et al., 1993 [31]	-	-
Sibbald et al., 2008 [32]	-	-
Van Hoof et al., 2016 [33]	-	-
**Author, year**	**Outcome measurement**	**Results**
**Costs**		
Black et al., 1997 [23]	a. Data from specialists were used to estimate subsequent costs of future appointment or treatment b. Managers in GP practice and in contract and finance departments in hospitals were asked to provide information for estimating health service costs per patient	a. With respect to treatment costs case-mix data suggest that dermatology and orthopaedics outreach patients and outpatients differed in the type and severity of their condition. The two groups were not comparable on costs b. Dermatology outreach clinics had significantly lower health service costs per patient. The average difference was £20.14. Marginal costs (the costs of treating an additional patient) were significantly higher for outreach clinics in both specialties. The average difference for dermatology: £4.17, and for orthopaedics: £9.59
Bond et al., 2000 [24]	Data on costs gathered via the patient survey and via managers from outreach clinics and accountants from outpatient hospital care. Outcome measurements: a. Personal costs (costs of travel, carers, child minders, time) b. Specialists’ travel costs and opportunity costs of travel time c. NHS staffing costs per clinic d. Patients’ treatment costs e. NHS fixed overhead costs	a. Outreach costs £4.55, outpatient costs £9.97 b. Outreach costs £15.52, outpatient costs £11.07 c. Outreach costs £13.80, outpatient costs £11.07 d. Outreach costs £163.73, outpatient costs £109.20 e. Not given since figures were not standardizable
Bowling and Bond, 2001 [25]	Costs were calculated per patient and per clinic type. a. Mean travel cost b. Mean opportunity cost c. Mean total costs to patients d. Mean NHS treatment costs e. Mean NHS staffing costs f. Mean total NHS costs	a. Outreach costs £0.82, outpatient costs £2.08 b. Outreach costs £3.09, outpatient costs £6.21 c. Outreach costs £3.96, outpatient costs £8.40 d. Outreach costs £135.21, outpatient costs £96.26 e. Outreach costs £14.38, outpatient costs £10.53 f. Outreach costs £149.59, outpatient costs £106.79
Bowling et al., 1997 [26]	Cost data were gathered using the self-administered patient questionnaire	Cost data analysis was still ongoing
Dart, 1986 [27]	For 46 patients in three months for whom a referral to the hospital was prevented costs were calculated in the eye community centre and costs if they were referred to hospital. The difference indicates the savings the community service provided	In three months’ time, the potential costs for the 46 patients in hospital would be £767.74 and the costs in the community health centre were £422.81. The conclusion of the paper is that the eye service in the community health centre saved £344.93 (minor savings)
Gillam et al., 1995 [28]	Measurements on: a. Staffing costs b. Travel costs of specialists c. Medication costs d. Overhead costs e. Depreciation of equipment costs f. Total costs g. Costs per patient	a. Outreach costs £368.80, outpatient costs £282.10 per session b. Outreach costs £14.10 per session c. Outreach costs £1.40, outpatient costs £6.74 per session d. Outpatient costs 11.03 per session (none for outreach) e. Outreach costs £10.04, outpatient costs £97.70 per session f. Outreach costs £394.34, outpatient costs £397.57 per session g. Per patient seen: outreach £48.09, outpatient £15.71
Gosden et al., 1997 [29]	A post-consultation questionnaire collected information on the patient’s travel and other out of-pocket expenses (e.g. the cost of paying a carer to look after dependants). Cost measurements of: a. Average travel costs for patients b. Cost for time spent at consultations c. Total patient costs d. Average staff costs per patient per clinic e. Average staff travel costs per patient per clinic f. Marginal costs per patient per clinic g. Overhead costs per patient per clinic h. Prescription costs per patient per clinic i. Test and investigation costs per patient per clinic j. Procedure costs per patient per clinic k. Total health service costs per patient per clinic	a. No significant results b. No significant results c. No significant results d. Dermatology outreach £6.27, outpatient £3.62 Orthopaedic outreach £9.60, outpatient £6.09 e. Dermatology outreach £1.52, outpatient not applicable (NA) Orthopaedic outreach £9.60, outpatient NA f. Dermatology outreach £7.79, outpatient £3.62 Orthopaedic outreach £15.68, outpatient £6.09 g. Dermatology outreach £2.78, outpatient £8.69 Orthopaedic no significant results h. Dermatology outreach £6.86, outpatient £11.69 Orthopaedic no significant results i. Dermatology outreach £5.75, outpatient £5.23 Orthopaedic no significant results j. Dermatology outreach £20.60, outpatient £34.69 Orthopaedic no significant results k. Dermatology outreach £43.78, outpatient £63.92 Orthopaedic no significant results
Helliwell, 1996 [30]	Measurements on: a. Costs per square foot of clinic space b. Staff costs (1/40th of monthly salary) c. Costs per patient	a. CC £18.50, HC £65 b. CC £114, HC £262 c. Costs per patient lower for HC than for CC due to higher number of patients attending the clinic. CC £15.93, HC £10.35
Little et al., 1993 [31]	Measurements on: a. Visit costs (not sure for whom)	a. A community clinic cost £23 compared to £37 for an outpatient visit
Sibbald et al., 2008 [32]	Outcome measurements on: a. Estimated cost per patient to commissioners	a.—For an orthopaedics service: Closer to home (CtHs) service £167.43, hospital service (HS) £289.86 - For a dermatology service: CtHs £74.61, HS £154.22 - For a gynaecology service: CtHs £237.90, HS £277.78
Van Hoof et al., 2016 [33]	-	-
**Author, year**	**Outcome measurement**	**Results**
**Substitution in joint consultation interventions**		
**Quality of care**		
Schulpen et al., 2003 [34]	A patient satisfaction survey was used to gather data on quality. No description of the type of survey. Outcome measurements were not defined	There was no significant difference in satisfaction between joint consultation patients and outpatients
Surís et al., 2007 [35]	A 5-item satisfaction questionnaire was given to the involved GPs. Outcome measurements: a. Mean waiting time for new non-urgent visits b. GP satisfaction with patient accessibility to the rheumatology unit in hospitals	a. The mean waiting time for new non-urgent rheumatology patients dropped by 15 days per person and month compared to the period before the intervention started b. Patient accessibility increased significantly according to GPs compared to the period before the intervention started
Vierhout et al., 1995 [36]	Only referrals to outpatient hospital care were used as a measurement of quality in terms of effective care	There was a significant difference in the degree to which patients of the joint consultation group were referred to outpatient hospital care after the consultation (18.8%) compared to the control group (32.0%)
**Author, year**	**Outcome measurement**	**Results**
**Health**		
Schulpen et al., 2003 [34]	Health status was measured via the EQ5D survey	There was no significant difference in health status at follow-up between joint consultation patients and outpatients
Surís et al., 2007 [35]	-	-
Vierhout et al., 1995 [36]	General health status was measured with a perceived state of health questionnaire (based on a Netherlands Central Statistics Bureau questionnaire)	There were no significant differences in the degrees of health improvement between the joint consultation and control groups. 35.4% of the joint consultation group was, however, symptom-free one year after the consultation compared to 23.7% of the control group
**Author, year**	**Outcome measurement**	**Results**
**Costs**		
Schulpen et al., 2003 [34]	Only referrals to outpatient hospital care were used as a proxy to measure costs	After two years, involved intervention GPs referred 62% fewer patients to outpatient hospital care than control GPs
Surís et al., 2007 [35]	Only referrals to outpatient hospital care were used as a proxy to measure costs	Compared to the period before the intervention started, 2.59% fewer patients were referred to the rheumatology outpatient units
Vierhout et al., 1995 [36]	-	-

Abbreviations: GP = general practitioner; CC = community clinic; HC = hospital clinic; HSQ-12 = health status questionnaire; DLQI = dermatology life quality index; NHS = national health; service; CtHs = closer to home service; EQ5D = EuroQol5D (questionnaire)

### Results per type of intervention

#### Substitution interventions in a (multidisciplinary) GP practice

Of the 14 included papers, 11 reported on the results of substitution interventions in a (multidisciplinary) GP practice. This type of intervention was described as a shift of hospital-based medical specialists to general practice settings without moving the facilities of the hospital to these settings, in order to prevent unnecessary referrals to outpatient hospital care [23–33]. The quality assessment of these papers resulted in no strong papers, five moderate papers [24–26, 32, 33] and six weak papers [23, 27–31]. The table with the quality assessment can be found in S1 Table. Of the 11 papers, nine concerned a cohort analytic study [23–26, 28–32], one concerned a cohort study in which the same group is pretested and tested after the intervention [27] and one paper was a case control study with a retrospective design [33].

The studies involved various medical specialties (see Table 1). Rheumatology was involved in six papers [24–26, 30, 34, 35]. Orthopaedics [23, 29, 32, 33], ear, nose and throat (ENT) [24–26, 32], dermatology [23, 29, 32, 33], internal or general medicine [24, 25, 32, 33] and gynaecology [24–26, 32] were involved in four papers. Ophthalmology was involved in three papers [27, 28, 31], cardiology in two papers [24, 25], and urology [32], paediatrics [25] and neurology [33] in one paper.

The 11 papers involved various data collection methods to measure *quality of care*. Self-administered questionnaires were used in nine studies to collect data on waiting list times, waiting times in the clinic, travelling times for patients, satisfaction with the received care, and follow-up after the consultation [23–26, 28–30, 32, 33]. Six of the papers reported shorter waiting list times for intervention patients than for outpatient hospital care patients when considering all medical specialties involved in the studies [23–25, 28, 29, 32]. In one paper they concluded that there were no significant differences between the two groups [26]. Waiting times in clinics were mostly shorter for intervention patients than for outpatient hospital care patients [24–26, 28, 30]. Only the study of Black et al. (1997) reported shorter waiting times in outreach clinics for orthopaedic patients and longer waiting times in outreach clinics for dermatology patients compared to the outpatient hospital care patients [23]. Two of the papers concluded that intervention patients had shorter travelling times than outpatient hospital care patients [23, 28]. Various aspects of patient satisfaction were studied in nine papers [23–26, 28–30, 32, 33]. Generally, intervention patients were more satisfied with aspects of the consultations than outpatient hospital care patients. Only one paper reported higher satisfaction rates for outpatient hospital care patients related to the time spent waiting at the clinic to see the specialist. This is the same paper that reported on longer waiting times in the clinic for dermatology outreach patients [23]. Five papers reported on follow-up after the consultation, and all concluded that more intervention patients were completely discharged after the consultation [24–26, 30, 31]. This effect was independent of patient case-mix differences (e.g. clinical severity) between the groups. Gillam et al. (1995) measured the ability of GPs to diagnose and manage medical conditions before and after the intervention period [28]. They concluded that GPs who spent time with the medical specialist for learning opportunities were better able to diagnose and manage the medical conditions mentioned in the study than GPs who did not spend time with the medical specialist.

Three of the 11 papers measured *health outcomes*, which were measured via the Health Status Questionnaire (HSQ-12) or the Dermatology Quality of Life Index (DQLI) [23–25]. All three concluded that there was a greater improvement of the perceived health or pain status for the intervention patients compared to the outpatient hospital care patients.

Lastly, *costs* were measured by ten of the 11 papers [23–32]. One reported that the cost analysis was still ongoing, and therefore reported in another article [26]. This, however, could not be found in databases or by checking reference lists. Five of the papers reported that generally speaking, the costs for patients were lower for intervention patients than for outpatient hospital care patients [23–25, 28, 29]. However, when analysing the total or marginal costs (the costs for treating an extra patient), outpatient hospital care is significantly cheaper than care in the intervention clinics [23–25, 28–32]. This is mainly caused by the higher patient numbers in outpatient hospital care than in the intervention clinics, which seemed to result in more efficient care. Only Dart (1986) said that the performance of a community service saved money during the intervention period [27]. It is, however, not clear how these costs were measured, and whether this was comparable to the measurements of the other papers that reported otherwise.

#### Joint consultation model

Of the 14 included papers, three involved a joint consultation model. This model is defined as an intervention in which medical specialists from outpatient hospital care perform joint consultations with GPs in a primary care setting to discuss medical cases and to agree on an approach of case management [34–36]. The quality assessment resulted in one strong paper [36], one moderate paper [34], and one weak paper [35]. Two papers were an RCT [34, 36] and one was a cohort study [35]. Rheumatology was involved in two papers and the other paper described orthopaedics.

Two papers measured *quality outcomes* via a patient questionnaire [34, 35]. In the paper by Schulpen et al. (2003), no details were described on how quality outcomes were measured. They only reported that there was no significant difference in satisfaction between the joint consultation patients and the outpatient hospital care patients [34]. In the paper of Surís et al. (2007), the mean waiting time for new non-urgent visits at the rheumatology department in the hospital and the GP satisfaction with patient accessibility to the rheumatology unit in the hospital were measured. GPs reported a better accessibility for patients and the mean waiting time decreased by 15 days per person compared to the period before the joint consultation intervention started [35]. The paper by Vierhout et al. (1995) measured quality in terms of effective care, by reporting on the percentage of patients that needed follow-up in outpatient hospital care after the joint consultation. Of the joint consultation patients, 18.8% needed follow-up outpatient hospital care, compared to 32.0% of the control group [36].

Two papers measured the *health status* of patients [34, 36]. Health status was measured with the European Quality of Life-5 Dimensions survey (EQ-5D) [34] and with a questionnaire based on a questionnaire from Statistics Netherlands (Centraal Bureau voor de Statistiek, in Dutch) [36]. Both papers reported that there was no significant improvement of the perceived health at follow-up between the joint consultation group and the usual outpatient hospital care group [34, 36]. Vierhout et al. (1995) stated, however, that a higher percentage of the joint consultation group was symptom-free one year after the intervention, compared to the control group [36].

To analyse the *cost outcome* of the joint consultation intervention, two of the three papers used referrals to outpatient hospital care as a proxy for cost savings [34, 35]. Schulpen et al. (2003) showed that after two years the involved intervention GPs referred 62% fewer patients to outpatient hospital care than the control GPs [34]. Compared to the period before the Spanish joint consultation programme, 2.59% fewer patients were referred to outpatient hospital care for all medical specialties in the whole population [35]. This referral rate was measured by dividing the number of referrals to the rheumatology department of all GPs in the involved area by the total number of the population seen by GPs during the study period. Both studies stated that the intervention caused fewer referrals to outpatient hospital care, and could therefore be more cost-effective, although the degree differed considerably.

## Discussion

The aim of this study was to provide a descriptive overview of papers in which substitution of outpatient hospital care with primary care-based interventions was performed by medical specialists in physical consultations in a primary care setting and the effects on the Triple Aim. The searching and selection process resulted in 1,389 studies, from which 14 papers were included in this review. Of these papers, 11 concerned substitution interventions in which medical specialists performed consultations in a (multidisciplinary) GP practice [23–33] and three papers concerned a joint consultation model [34–36].

The quality assessment resulted in one strong paper, six moderate papers and seven weak papers. This is mainly due to the fact that the quality assessment tool is relatively strict about the advantage of randomization in the light of preventing (selection) bias. In only three papers the subjects were randomly selected. It is, however, difficult to find a qualitative good quality assessment tool that is not strict, or less strict, on randomization [37]. Besides, an RCT can only be performed if all (surrounding) conditions can be held constant. The types of interventions studied in this systematic review, are difficult to control and to hold constant. Additionally, often it is impossible and undesirable to control and hold constant a real-life situation [38].

This systematic review showed a wide variety of studied medical specialties in substitution interventions. ENT, dermatology, orthopaedics, internal medicine, gynaecology and rheumatology were mostly involved. Neurology and urology were only involved in one paper and cardiology in two papers. The paper of Van Hoof et al. (2016), for example, stated that cardiologists needed more diagnostic equipment than available in GP practices, and were therefore not able to perform the intervention properly [33].

The *quality outcome* should be measured according to at least one of the IOM guidelines (safety of care, effectiveness, patient-centredness, timeliness, efficiency and/or equitability) [19]. None of the papers that measured quality had outcome measures in terms of safety or equitable care. One explanation could be that both are difficult to operationalize. Almost all papers reported shorter waiting list times, shorter waiting times in the GP practices, higher patient satisfaction and fewer follow-up visits after the consultation in the primary care setting [23–26, 28–33, 35]. Only one paper mentioned a favourable outcome for the outpatient hospital care patients, which was related to the waiting time in the clinic [23]. These findings suggest substitution did not always result in shorter waiting times in the clinic and it is therefore important to focus on providing short waiting lists and short waiting times at the clinic to be able to accomplish high-quality care. Another precondition for the studied types of substitution interventions should be the opportunity for GPs to learn from medical specialists. The one paper that reported on this issue concluded that GPs who took the opportunity to deliberate with medical specialists were better able to diagnose medical conditions than GPs who did not take this opportunity [28]. Also, the joint consultation model, in which GPs were able to discuss patient cases with a medical specialist, resulted in more effective care [36]. A qualitative study on the preconditions for Primary Care Plus, an intervention in which medical specialists perform consultations in GP practices, showed that both GPs and medical specialists considered it very important to have the opportunity to deliberate. GPs indicated that they would like to learn from medical specialists, to be better able to diagnose and help similar patients in the future [39].

In relation to the *health outcomes*, only five papers measured health status after the consultation in the primary care setting compared to outpatient hospital care [23–25, 34, 36]. Three papers reported higher health status at follow-up for the intervention patients [24, 25, 36], one paper concluded that there was a greater health improvement for dermatology control group patients, and a greater health improvement for orthopaedic intervention patients [23], and one paper concluded that there were no differences between the two groups [34].

*Cost outcomes* were measured in 11 of the 14 papers [23–25, 27–32, 34, 35]. Papers that reported on cost outcomes, which were calculated in money savings, concluded that the costs for facilities and the costs for patients were lower in the intervention settings than in outpatient hospital care [23–25, 28–30]. In contrast, the staffing and marginal costs were higher for the interventions in the primary care setting [24, 25, 28, 32]. Two other papers used the referrals to outpatient hospital care after the consultation as a proxy for costs [34, 35]. They concluded that GPs referred significantly fewer patients to outpatient hospital care after the intervention period. Overall, when looking at the papers that involved real costs, it seemed that this review could conclude that the costs for helping an extra patient are higher in interventions in primary care settings, mainly due to the lower number of patients seen. The adherence area should therefore be (very) large to be cost-effective compared to outpatient hospital care in which many more patients could be served. Additionally, the consultation hours should be planned efficiently to ensure efficient use of the medical specialists’ time.

Internationally, a lot is written about the importance of performing substitution interventions which aim to prevent unnecessary referrals to outpatient hospital care because of the unlimited increasing healthcare costs [1–3]. The expectation, therefore, was that this review would uncover many relevant papers. However, only 14 papers were included, of which eight were published before the millennium [23, 26–31, 36]. One explanation could be that these kinds of initiatives are difficult to study in terms of quality, health and costs. And therefore there is a lack of generalizable data about the costs and effectiveness of substitution initiatives. A recent Dutch article stated that substitution in health care is a transitional process that should be monitored continuously in a longitudinal study design [40]. The problem many researchers encounter is that to prove that an intervention is effective in terms of quality, health and costs, regional, or nationwide data are needed over a long period of time. One should be cautious in stating that referrals to a hospital decreased in a certain period of time. In order to prove a decrease in referrals, one should be certain that these patients were not seen in another institution in an outpatient hospital care setting instead. Additionally, to capture a learning effect for involved GPs, longitudinal data are also necessary.

It is remarkable that five of the 14 included papers in this review are from the same group of authors (Black, Bond, Bowling and Gosden) [23–26, 29]. These five papers concerned substitution of outpatient hospital care with primary care in the so-called outreach clinics. The advantage of this, however, is that these interventions are evaluated in a somewhat similar manner, and therefore the outcomes of these papers are comparable to each other. Furthermore, it is also remarkable that the included papers originated from only three different countries, namely the United Kingdom, the Netherlands and Spain. These countries have a long tradition of a strong primary-care-based system with a gatekeeping role for the GP, which means that hospital-, and specialist care is only accessible with a referral of the GP (except for emergency care) [41]. This could have influenced the results of this review; countries with a less strong primary care system and/or no gatekeeping system are probably less focused on substitution of care initiatives such as providing specialist care in the primary care setting. However, most Scandinavian countries also have strong primary care systems and this literature search did not find any published studies in these countries.

One limitation of this study is the narrow scope of the interventions relevant for this systematic review. A substitution intervention should be performed by medical specialists and not by other healthcare providers, in face-to-face consultations in a primary care setting. There are, however, studies about substitution interventions in which outpatient hospital care is substituted with care performed by other healthcare providers, such as a ‘new’ profession like a nurse practitioner, or through e-consults [42–44]. Though, a qualitative study on the preconditions of Primary Care Plus showed that the profile of the medical specialist was particularly important for the success of such an intervention [39]. GPs indicated that they were more likely to refer patients to an experienced medical specialist, who supports the substitution idea, than to other healthcare providers.

## Conclusions

Despite the widely held idea that substitution of outpatient hospital care with primary care contributes to more efficient and cost-effective healthcare systems, only a few papers exist in which medical specialists perform consultations in primary care settings. However, this study showed that substitution initiatives in which medical specialists perform consultations in primary care settings to prevent unnecessary referrals to outpatient hospital care result mostly in favourable outcomes compared to usual outpatient hospital care. The initiatives involved resulted mostly in shorter waiting lists, shorter waiting times in clinics and higher patient satisfaction than in outpatient hospital care. Costs for treating one extra patient, however (the marginal costs), seemed to be higher in the intervention settings than in outpatient hospital care. This was mainly caused by inefficient planning of consultation hours and lower patient numbers referred to the interventions. Future (adaptations of) interventions should therefore focus on an adherence area that is large enough to fill the consultation hours efficiently. Future systematic reviews should focus on the effects on the Triple Aim of substitution initiatives in which nurse practitioners and other healthcare professions are also involved, along with new technologies, like e-consults. Above all, to receive more insight into the effects of substitution initiatives operating in a dynamic healthcare context, it is important to keep evaluating the interventions in a longitudinal study design.

## Supporting information

S1 File Search strategy. (DOCX)

S2 File PRISMA 2009 checklist. (DOCX)

S1 Table Quality assessment of included studies. (DOCX)

S2 Table Extensive version Table 1. (DOCX)
