# Supplementary material for: Substitution of outpatient hospital care with specialist care in the primary care setting: A systematic review on quality of care, health and costs
Source: PLoS One. 2019 Aug 1;14(8):e0219957. doi: 10.1371/journal.pone.0219957 (PMC6675042; doi:10.1371/journal.pone.0219957)
Supplement: S1 File — (DOCX) [file pone.0219957.s001.docx]

**Search strategy**

(((((((((((("joint consultation"[All Fields] OR "joint consultations"[All Fields]) OR "outreach"[All Fields]) OR "outreach clinic"[All Fields]) OR "outreach clinics"[All Fields]) OR "outreach service*"[All Fields]) OR "specialist clinic*"[All Fields]) OR "specialist service*"[All Fields]) OR "substitution"[All Fields]) OR (visiting[All Fields] AND ("referral and consultation"[MeSH Terms] OR ("referral"[All Fields] AND "consultation"[All Fields]) OR "referral and consultation"[All Fields] OR "consultation"[All Fields]))) OR "visiting service"[All Fields]) AND (((("primary health care"[MeSH Terms] OR ("primary"[All Fields] AND "health"[All Fields] AND "care"[All Fields]) OR "primary health care"[All Fields] OR ("primary"[All Fields] AND "healthcare"[All Fields]) OR "primary healthcare"[All Fields]) OR ("general practice"[MeSH Terms] OR ("general"[All Fields] AND "practice"[All Fields]) OR "general practice"[All Fields])) OR ("general practitioners"[MeSH Terms] OR ("general"[All Fields] AND "practitioners"[All Fields]) OR "general practitioners"[All Fields] OR ("general"[All Fields] AND "practitioner"[All Fields]) OR "general practitioner"[All Fields])) OR ("family practice"[MeSH Terms] OR ("family"[All Fields] AND "practice"[All Fields]) OR "family practice"[All Fields]))) AND (((((((("costs and cost analysis"[MeSH Terms] OR ("costs"[All Fields] AND "cost"[All Fields] AND "analysis"[All Fields]) OR "costs and cost analysis"[All Fields]) OR "costs"[All Fields]) OR ("health care costs"[MeSH Terms] OR ("health"[All Fields] AND "care"[All Fields] AND "costs"[All Fields]) OR "health care costs"[All Fields])) OR ("health expenditures"[MeSH Terms] OR ("health"[All Fields] AND "expenditures"[All Fields]) OR "health expenditures"[All Fields] OR ("health"[All Fields] AND "expenditure"[All Fields]) OR "health expenditure"[All Fields])) OR "expenditures"[All Fields]) OR ((("health outcomes"[All Fields] OR "experienced health"[All Fields]) OR "disease burden"[All Fields]) OR ("quality of life"[MeSH Terms] OR ("quality"[All Fields] AND "life"[All Fields]) OR "quality of life"[All Fields]))) OR ((((((((((("patient satisfaction"[MeSH Terms] OR ("patient"[All Fields] AND "satisfaction"[All Fields]) OR "patient satisfaction"[All Fields]) OR "patient experiences"[All Fields]) OR "experience of care"[All Fields]) OR ("personal satisfaction"[MeSH Terms] OR ("personal"[All Fields] AND "satisfaction"[All Fields]) OR "personal satisfaction"[All Fields])) OR "satisfaction"[All Fields]) OR "patient safety"[All Fields]) OR "treatment effectiveness"[All Fields]) OR ("patient-centered care"[MeSH Terms] OR ("patient-centered"[All Fields] AND "care"[All Fields]) OR "patient-centered care"[All Fields] OR ("patient"[All Fields] AND "centered"[All Fields] AND "care"[All Fields]) OR "patient centered care"[All Fields])) OR "efficient care"[All Fields]) OR "timely care"[All Fields]) OR "equitable care"[All Fields])) OR "triple aim"[All Fields])) AND ("1985/01/01"[PDAT] : "2017/03/16"[PDAT])
