# Supplementary material for: Substitution of outpatient hospital care with specialist care in the primary care setting: A systematic review on quality of care, health and costs
Source: PLoS One. 2019 Aug 1;14(8):e0219957. doi: 10.1371/journal.pone.0219957 (PMC6675042; doi:10.1371/journal.pone.0219957)
Supplement: S1 Table — (DOCX) [file pone.0219957.s003.docx]

**Quality assessment of included studies**

| **Author** | **Year** | **1) Selection bias** | **2) Study design** | **3) Confounders** | **4) Blinding** | **5) Data collection methods** | **6) Withdrawals**  **and drop-outs** | **Global rating** |
| --- | --- | --- | --- | --- | --- | --- | --- | --- |
| Black et al. | 1997 | W | M | W | M | S | M | Weak |
| Bond et al. | 2000 | W | M | S | M | S | M | Moderate |
| Bowling et al. | 2001 | W | M | S | M | S | M | Moderate |
| Bowling et al. | 1996 | W | M | S | M | S | NP | Moderate |
| Dart | 1986 | W | M | W | M | W | NP | Weak |
| Gillam et al. | 1995 | W | M | W | M | W | M | Weak |
| Gosden et al. | 1997 | W | M | S | M | W | NP | Weak |
| Helliwel | 1996 | W | M | W | M | W | M | Weak |
| Little, et al. | 1993 | S | W | W | M | S | NP | Weak |
| Schulpen, et al. | 2003 | M | S | S | M | S | NP | Moderate |
| Sibbald, et al. | 2008 | W | M | S | M | M | NP | Moderate |
| Surís, et al. | 2007 | W | M | W | M | W | W | Weak |
| Van Hoof et al. | 2016 | W | M | S | M | S | NP | Moderate |
| Vierhout et al. | 1995 | S | S | S | M | S | M | Strong |

S = Strong

M = Moderate

W = Weak

NP = Not applicable
