# Supplementary material for: Substitution of outpatient hospital care with specialist care in the primary care setting: A systematic review on quality of care, health and costs
Source: PLoS One. 2019 Aug 1;14(8):e0219957. doi: 10.1371/journal.pone.0219957 (PMC6675042; doi:10.1371/journal.pone.0219957)
Supplement: S2 Table — (DOCX) [file pone.0219957.s004.docx]

**Extensive version Table 1**

**Table 1 Characteristics of included studies**

| **Author(s), year** | **Country** | **Type of intervention** | **Medical specialty(ies) involved** | **Study design** | **Sample characteristics** | | | **Control group** |
| --- | --- | --- | --- | --- | --- | --- | --- | --- |
|  |  |  |  |  | **N** | **Age (mean)** | **Gender**  **(% male)** |  |
| Black et al., 1997 | United Kingdom | Outreach clinics | Dermatology and orthopaedics | Cohort analytic study (case-referent comparative study)  Aim: to describe outreach clinics from perspectives of patients, GPs and medical specialists and comparison of costs. Data collected through questionnaires and cost data supplied by managers. | 164 patients  3 dermatologists  3 orthopaedics  6 GPs | x | x | Patients who visited the dermatology or orthopaedics department in outpatient hospital care. |
| Bond et al., 2000 | United Kingdom | Outreach clinics | Cardiology, ENT, general medicine, general surgery, gynaecology and rheumatology | Cohort analytic study (case-referent comparative study).  Aim: to measure processes of care, health benefits and costs of outreach clinics compared to outpatient hospital care. Data collected through self-administrated questionnaires for patients, medical specialists and GPs. | 1420 patients  18 medical specialists  54 GPs | 7% < 16 years old  4% 16<25 years old  31% 25<45 years old  35% 45<65 years old  23% ≥ 65 years old | 32% | Patients who visited the involved medical departments in outpatient hospital care. |
| Bowling et al.,  2001 | United Kingdom | Outreach clinics | Cardiology, ENT, general medicine, general surgery, gynaecology, paediatrics and rheumatology | Cohort analytic study (case-referent comparative study).  Aim: to evaluate costs, processes and benefits of outreach clinics compared to outpatient hospital care.  Self-administrated questionnaires for patients, medical specialists and GPs. | 2925 patients  38 medical specialists  196 GPs | 9% - < 16 years  5% - 16 -25 years  30% - 25 - 45 years  33% - 45 - 65 years  23% - > 65 years | 31 | Patients who visited the involved medical departments in outpatient hospital care. |
| Bowling et al., 1996 | United Kingdom | Outreach clinics | ENT, rheumatology and gynaecology | Cohort analytic study (case-referent comparative study).  Aim: to collect data on processes of outreach clinics compared to outpatient hospital care.  Self-administrated questionnaires for patients, medical specialists and GPs. And data collected from hospital system. | 146 patients  9 practice managers  9 medical specialists  60 GPs | x | x | Patients who visited the involved medical departments in outpatient hospital care. |
| Dart, 1986 | United Kingdom | Ophthalmologist consulted patients in a community centre. | Ophthalmology | Cohort study. 30 Patients were first seen by a GP and were then asked to be consulted by an ophthalmologist. The diagnoses of the GPs and the ophthalmologist were compared to each other. Differences in diagnosis were measured, costs were measured for the group of patients for which a referral to outpatient hospital care was prevented in the community health centre and in the case they were referred to outpatient hospital care. | 46 patients | x | x | Diagnosis of GP was compared to diagnosis of ophthalmologist |
| Gillam et al., 1995 | United Kingdom | Outreach clinics | Ophthalmology | Cohort analytic study (case-referent comparative study).  Aim: to evaluate outreach in ophthalmology of its impact on GPs, use of secondary care services, patients’ views and costs. Referral data is collected. GPs received a survey. And patient surveys were conducted using self-administered questionnaires. | 1309 patients  63 GPs | x | x | Patients who visited the ophthalmic department in outpatient hospital care. |
| Gosden et al., 1997 | United Kingdom | Outreach clinics | Dermatology and orthopaedics | Cohort analytic study (case-referent comparative study).  To study the cost-effectiveness of the outreach clinics. Information was collected on waiting times, costs to patients, case mix and resource use. | 242 patients | x | x | Patients who visited the dermatology or orthopaedics department in outpatient hospital care. |
| Helliwel, 1996 | United Kingdom | Rotating community clinic with involvement of consultant specialists | Rheumatology | Cohort analytic study (case-referent comparative study).  To provide an evaluation of the community clinic in terms of descriptive consultation data, patient satisfaction, case mix and costs. Data was collected from a database and a patient survey. | 135 patients | x | 30.4 | Patients who visited the rheumatology department in outpatient hospital care. |
| Little, et al., 1993 | United Kingdom | Outreach clinics | Ophthalmology | Cohort study | 126 patients of 75 years and older | x | x | No control group |
| Schulpen et al., 2003 | the Netherlands | Joint consultation | Rheumatology | Randomized controlled trial.  To compare the effects of joint consultation in rheumatology on health status, referral rates and care policy with usual outpatient hospital care. Data collected through questionnaires for medical specialists, GPs and patients. | 166 patients  6 rheumatologists  17 GPs | 53.7 | 27 | Patients who visited the rheumatology department in outpatient hospital care. |
| Sibbald, et al., 2008 | United Kingdom | Specialist care in the community | Dermatology, ENT, general surgery, gynaecology, orthopaedics and urology | Cohort analytic study. To describe service organization and the factors which facilitated or impeded service development; estimate the likely impact on local health economies; and assess patients’ views about service access, quality and coordination. Interviews and questionnaires are conducted. | 58 Service managers, GPs, care providers and medical specialists  1,233 Patients | x | x | Patients who visited the outpatient hospital care departments. |
| Surís, et al., 2007 | Spain | Rheumatology consultancy programme | Rheumatology | Cohort study. To analyse the influence of a primary care rheumatology consultancy programme on the number of referrals to the rheumatology unit in outpatient hospital care, the waiting times for new visits, and the satisfaction of the GPs with respect to the rheumatology unit. | 120 consultancy session cases  117 patients | x | x | No control group |
| Van Hoof et al., 2016 | the Netherlands | Primary Care Plus | Internal medicine, dermatology, orthopaedics and neurology | Case control study. Patient experience was measured in intervention and control group. | 78 patients in intervention group  104 patients in control group | Intervention group 54.7  Control group 53.8 | Intervention group 41.5  Control group 38.6 | Patients who visited the internal medicine, dermatology, orthopaedics and neurology department in outpatient hospital care. |
| Vierhout et al., 1995 | the Netherlands | Joint consultation | Orthopaedics | Randomized controlled trial.  To study the effects of joint consultation in orthopaedics on referral and intervention rates. Data on symptoms were collected through a patients questionnaire, additional medical data was recorded by the GPs. | 272 patients | Intervention group 16% - > 60 years  Control group  13% - > 60 years | Intervention group - 49%  Control group - 50% | Patients who visited the orthopaedics department in outpatient hospital care. |
